# From trees to 3D printing: “all-wood” photopolymer composites based on bisguiacol-F-diacrylate and methacrylated pinewood flour for digital light processing (DLP)

**DOI:** 10.1039/d6py00486e

**Published:** 2026-06-11

**Authors:** Ettore Greco, Nicola Porcelli, Matteo Bergoglio, Niccolò Braidi, Fabrizio Roncaglia, Marco Sangermano, Minna Hakkarainen

**Affiliations:** a Department of Applied Science and Technology (DISAT), Politecnico di Torino Torino 10129 Italy marco.sangermano@polito.it; b Department of Chemical and Geological Sciences, Università degli studi di Modena e Reggio Emilia Modena 41125 Italy; c KTH Royal Institute of Technology, Department of Fibre and Polymer Technology Teknikringen 58 100 44 Stockholm Sweden

## Abstract

A fully wood-derived (“all-wood”), high-performance photocurable resin has been developed specifically for room-temperature vat 3D printing under low light intensity. The formulations were based on Bisguiacol-F-Diacrylate (DA), a lignin-derived aromatic monomer, and Acrylated Guaiacol (MA) employed as a reactive diluent. To enhance the mechanical and thermomechanical properties, the rheologically optimized formulation (70DA-30MA) was reinforced with Methacrylated Pinewood flour (MP) up to a loading of 10 phr. Rheological analysis confirmed a suitable viscosity for room-temperature printing, with the addition of MP inducing a shear-thinning behaviour. UV-curing kinetics were studied *via* FT-IR and Photo-DSC to investigate the effect of reactive diluent and filler addition. Morphological analyses (SEM) of the fracture surfaces of 3D-printed samples revealed excellent interfacial adhesion between the polymer matrix and wood flour, thanks to its surface functionalization. Thermal and mechanical properties of composite samples were investigated through DMTA and tensile tests, revealing an increase in the glass transition temperature (*T*_g_) from 38 °C to 62 °C, accompanied by an increase in Young's modulus and ultimate tensile strength (UTS). These results demonstrate the potential of biomass-derived aromatic monomers and reactive fillers for the fabrication of fully bio-based, advanced 3D composites, bridging the performance gap with traditional fossil-based resins.

## Abstract 2

An all-wood photocurable resin based on lignin-derived monomers were used in 3D printing and Reinforced with methacrylated pinewood flour.

## Graphical abstract

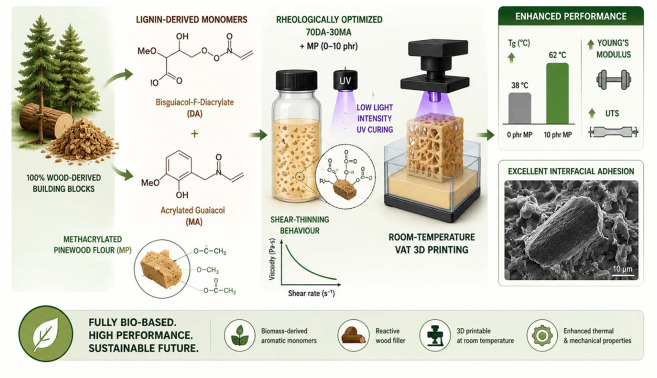

## Introduction

Additive manufacturing (AM) technologies based on light-induced polymerization, such as digital light processing (DLP), and stereolithography (SLA), have experienced rapid development. These vat photopolymerization techniques have the ability to fabricate complex geometries with high resolution and surface quality.^1–3^ They rely predominantly on photocurable resins composed of multifunctional (meth)acrylate monomers and oligomers, which undergo fast radical polymerization upon light exposure. Despite their technological maturity, the vast majority of commercially available photopolymer resins are derived from petroleum-based resources, raising increasing concerns regarding environmental impact, toxicity, and long-term sustainability. Consequently, the development of bio-based alternatives for photopolymerizable systems has become a critical research priority.^4–13^ In recent years, significant efforts have been directed toward the design of renewable photopolymers derived from biomass feedstocks, including plant oils,^9,13–15^ lignin derivatives,^16–19^ vanillin,^20–22^ and isosorbide-based compounds.^23,24^ These materials offer advantages such as reduced carbon footprint, renewability, and potential biodegradability. However, the transition from fossil-based to bio-based resins remains challenging, as many bio-derived monomers exhibit inferior performance in terms of mechanical strength, thermal stability, and curing efficiency. In particular, achieving the stringent requirements of light-induced AM, such as rapid curing kinetics, appropriate viscosity, and high crosslink density, remains a major limitation for bio-based formulations. Among the emerging candidates, aromatic bio-based monomers derived from lignin-related compounds have attracted considerable attention due to their intrinsic rigidity and potential to enhance thermomechanical properties. In this context, bisguaiacol-F-diacrylate represents a promising alternative to conventional bisphenol-A (BPA) based acrylates.^25,26^ Moreover, it has recently been found to be one of the best lignin derived dimers for BPA replacement, thanks to its non-oestrogenic trait.^27^ Synthesized from guaiacol, a lignin-derived phenolic compound, it can be easily functionalized with acrylates functional groups suitable for free-radical photopolymerization. The presence of aromatic moieties in its structure is expected to increase stiffness and thermal resistance of the resulting polymer network. While such characteristics make bisguaiacol-F-diacrylate an attractive candidate for sustainable photopolymers, its application in light-induced additive manufacturing remains largely underexplored, particularly in terms of printability, curing behavior, and final material performance. One of the major limitations of lignin-derived aromatic resins is their intrinsically high viscosity, a challenge that severely restricts the 3D printability, particularly at room temperature (RT). To overcome this drawback, the use of a reactive diluent is required. While various monomer combinations have been explored in the literature to address this issue,^25,26,28^ Acrylated Guaiacol (MA) was specifically selected for this study. This choice is justified by its close structural similarity to the primary diacrylate monomer and its shared origin as a lignin derivative, so that the overall environmental sustainability of the formulations is preserved. To further improve the mechanical and thermomechanical performance of photocured 3D-printed structure, the incorporation of bio-based reinforcements is reported as an effective strategy to enhance mechanical properties without compromising processability.^9,29–31^ In this study, Methacrylated Pinewood flour (MP) was added to ultimately target a high-performance “all-wood” polymer composite (WPC). Wood flour was specifically chosen as it is a direct byproduct of the woodworking industry, essentially comprising of finely pulverized wood, derived from abundant and renewable lignocellulosic biomass. Also, thanks to its high stiffness, low density, and large specific surface area it turns out to be particularly appealing, and it has been extensively investigated as a filler in previous studies.^32,33^ Nevertheless, its hydrophilic nature and tendency to agglomerate pose significant challenges when dispersing it within hydrophobic matrices. Surface functionalization has therefore been widely employed to improve compatibility as well as to enhance polymer network-filler interactions to maximize the reinforcing effect.^9,32^ Methacrylation of wood flour enables their covalent integration into the polymer network during photopolymerization, leading to improved dispersion, interfacial adhesion, and stress transfer efficiency. Despite growing interest in sustainable photopolymers, there remains a lack of comprehensive studies combining bio-based aromatic monomers, such as DA, with reactive fillers like MP in the context of light-induced AM. In particular, the interplay between resin composition, photopolymerization kinetics, printability, and the resulting mechanical performance of 3D-printed structures has not been fully elucidated. Within this framework, a bio-based photocurable system is developed based on DA as a polymeric precursor, combined with MA as a reactive diluent to modulate viscosity and with MP as reinforcing agent. The DA monomer is synthesized and formulated into UV-curable resins suitable for light-induced AM. The effect of MP incorporation on photopolymerization behaviour, printability, and the mechanical performance of the resulting 3D-printed structures is systematically investigated. This study aims to bridge the gap between sustainability and high performance in photopolymer-based additive manufacturing by proposing a fully bio-based, reinforced material system.

## Experimental

### Materials

All reagents and solvents were used as purchased without further purification. Grade of employed reagents was as follows: guaiacol (Gua, Sigma-Aldrich, 99%), ethylene carbonate (EC, Fluka, >99%), silica gel (Millipore, 0.063–0.200 mm mesh), dichloromethane (DCM, Sigma-Aldrich, 99.5%), ethyl acetate (EtOAc, Sigma-Aldrich, 99.5%), diethyl ether (Et_2_O, Sigma-Aldrich, 98%), methanol (MeOH) (Sigma-Aldrich, 99%), K_2_CO_3_ (Riedel-de Haen, >99%), H_2_SO_4_ (Scharlau, 95–97%), Na_2_SO_4_ (Sigma Aldrich, >99.0%). Phenylbis(2,4,6-trimethylbenzoyl)phosphine oxide (BAPO – Sigma-Aldrich, 97%) was employed as photoinitiator.

**Fig. 1:**
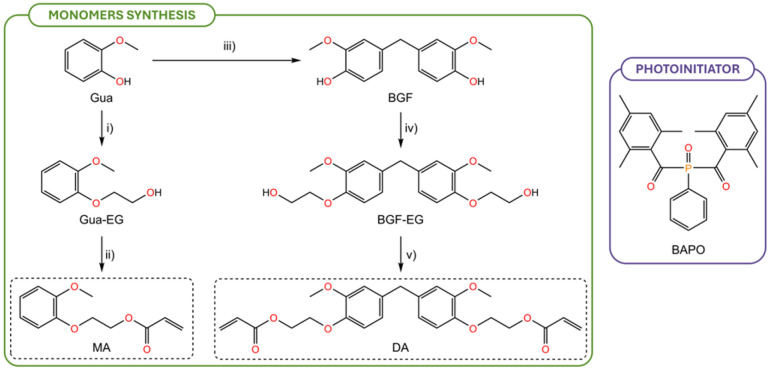
Reported synthetic pathways from guaiacol to its corresponding acrylate monomers^34^ and photoinitiator structure. Monomer synthesis: (i) EC (1.2 eq.), K_2_CO_3_ (0.1 eq.), 120 °C, 2 h; (ii) acryloyl chloride (1.2 eq.), triethylamine (1.5 eq.), 0 °C for 1 h, then RT for 2 h; (iii) vanillyl alcohol (0.125 eq.), SSANa 3.0 mmol g^−1^ (20% mol), 80 °C, 3 h; (iv) EC (2.4 eq.), K_2_CO_3_ (0.1 eq.), 120 °C, 2 h; (ii) acryloyl chloride (2.4 eq.), triethylamine (3.0 eq.), 0 °C for 1 h, then RT for 2 h.

BGF (bisguaiacol F, Fig. 1) was synthesized using a previously reported method.^34^ In this instance, chromatographic purification was not performed.

^1^H NMR (600 MHz, CDCl_3_) *δ* (ppm): 6.97–6.47 (m, 6H, Ar–H), 5.67–5.34 (bs, 2H, Ar–OH), 3.94–3.72 (m, 8H, Ar–O–CH_3_ and Ar–CH_2_–Ar). See also Fig. S1.

Gua-EG (2-(2-methoxyphenoxy)ethan-1-ol, Fig. 1) was synthesized adapting a procedure from the literature.^34^ Guaiacol (100 mmol), EC (120 mmol) and K_2_CO_3_ (10 mmol) were inserted into a 250 mL round bottom flask and heated to 120 °C for 2 h under magnetic stirring. Upon reaction completion, monitored by thin layer chromatography (TLC, eluent DCM : MeOH = 90 : 10), the mixture was quenched with aqueous H_2_SO_4_ (2 M, 5.0 mL), then diluted with EtOAc (150 mL) and transferred into a separatory funnel. Once removed the aqueous phase, the organic phase was washed with brine (5 × 100 mL) and dried over anhydrous Na_2_SO_4_. Removal of the volatiles under reduced pressure afforded the crude Gua-EG as a dark red liquid in quantitative yield, which was used without additional purification.

^1^H NMR (400 MHz, CDCl_3_) *δ* (ppm): 7.00–6.87 (m, 4H, Ar–H), 4.12 (t, *J* = 4.4 Hz, 3H, Ar–O–CH_2_), 3.92 (t, *J* = 4.8 Hz, 3H, C–CH_2_–O), 3.86 (s, 3H, Ar–O–CH_3_). See also Fig. S2.

^13^C NMR (100.61 MHz, CDCl_3_) *δ* (ppm): 150.1, 148.2, 122.3, 121.2, 115.3, 112.0, 71.6, 61.4, 55.9. See also Fig. S3.

BGF-EG (2,2′-((methylenebis(2-methoxy-4,1-phenylene))bis(oxy))bis(ethan-1-ol) and related isomers, Fig. 1) was synthesized adapting a previously known procedure.^34^ BGF (100 mmol), EC (240 mmol) and K_2_CO_3_ (10 mmol) were inserted into a 250 mL round bottom flask and heated at 120 °C for 2 h under magnetic stirring. Upon reaction completion, monitored by TLC (eluent DCM : MeOH = 90 : 10), the mixture was quenched with aqueous H_2_SO_4_ (2 M, 5 mL), then diluted with EtOAc (150 mL) and transferred into a separatory funnel. The organic phase was washed with brine (5 × 100 mL) and dried over anhydrous Na_2_SO_4_. Removal of the volatile fraction under reduced pressure afforded the crude BGF-EG as pale yellow solid in quantitative yield, which was used without additional purification.

^1^H NMR (400 MHz, CDCl_3_) *δ* (ppm): 6.89–6.62 (m, 6H, Ar–H), 4.12–4.09 (m, 4H, Ar–O–CH_2_), 3.91–3.88 (m, 4H, C–CH_2_–O), 3.88–3.72 (m, 8H, Ar–O–CH_3_ and Ar–CH_2_–Ar). See also Fig. S4.

MA (Acrylated Guaiacol, Fig. 1) was synthesized through acrylation of crude Gua-EG by an adaptation of an already known procedure.^34^ Purification through flash column chromatography over silica gel using Et_2_O as eluent, afforded MA as a yellow liquid in quantitative yield.

^1^H NMR (400 MHz, CDCl_3_) *δ* (ppm): 7.00–6.87 (m, 4H, Ar–H), 6.43 (dd, *J*_1_ = 1.2 Hz, *J*_2_ = 16.8 Hz, 1H, C=CH_2_), 6.17 (dd, *J*_1_ = 10.4 Hz, *J*_2_ = 17.4 Hz, 1H, C(=O)–CH=C), 5.84 (dd, *J*_1_ = 1.2 Hz, *J*_2_ = 10.4 Hz, 1H, C=CH_2_), 4.53 (t, *J* = 4.8 Hz, 2H, Ar–O–CH_2_), 4.28 (t, *J* = 5.2 Hz, 3H, C–CH_2_–O–C(=O)), 3.86 (s, 3H, Ar–O–CH_3_). See also Fig. S5.

^13^C NMR (100.61 MHz, CDCl_3_) *δ* (ppm): 166.3, 150.1, 148.1, 131.3, 128.3, 122.2, 121.1, 115.0, 112.4, 67.5, 63.2, 56.1. See also Fig. S6.

DA (bisguaiacol-F-diacrylate, Fig. 1) was synthesized through acrylation of crude BGF-EG, using an already known procedure.^34^ Purification through flash column chromatography over silica gel using Et_2_O as eluent, afforded DA as a yellow liquid in quantitative yield.

^1^H NMR (600 MHz, CDCl_3_) *δ* (ppm): 6.88–6.63 (m, 6H, Ar–H), 6.42 (dd, *J*_1_ = 1.2 Hz, *J*_2_ = 17.4 Hz, 2H, C=CH_2_), 6.16 (dd, *J*_1_ = 10.8 Hz, *J*_2_ = 17.4 Hz, 2H, C(=O)–CH=C), 5.84 (dd, *J*_1_ = 1.8 Hz, *J*_2_ = 15.6 Hz, 2H, C=CH_2_), 4.50 (t, *J* = 5.4 Hz, 2H, Ar–O–CH_2_), 4.25 (t, *J* = 4.8 Hz, 3H, C–CH_2_–O–C(=O)), 3.89–3.77 (m, 8H, Ar–O–CH_3_ and Ar–CH_2_–Ar). See also Fig. S7.

Methacrylated Pinewood flour (MP) was synthesized as reported in literature^32^ and manually grinded within a ceramic mortar before use. Fig. S8 shows SEM images of MP before and after grinding.

### Design of formulations

DA was mixed with MA in a ratio of 85 : 15 and 70 : 30 DA : MA to reduce the viscosity and make the formulation in a printable viscosity range. In each formulation, 2 phr (parts per hundred resin) of photoinitiator BAPO were added and sonicated for 15 minutes at 40 °C. Additionally, 70DA-30MA formulation was mixed with different amounts of MP filler (5, 7.5 and 10 phr – Table 1), the formulations were mixed using Ultraturrax set at 5000 rpm and then sonicated for 15 minutes at 40 °C.

**Table 1:** Composition of the photocurable formulations

Formulation	DA (wt%)	MA (wt%)	BAPO (phr)	MP (phr)
100DA	100	0	2	0
85DA-15MA	85	15	2	0
70DA-30MA	70	30	2	0
70DA-30MA_5MP				5
70DA-30MA_7.5MP				7.5
70DA-30MA_10MP				10

### Characterization methods

#### Viscosity measurements

To evaluate the viscosity of the photocurable formulations, rheological measurements were conducted using an Anton Paar Modular Compact Rheometer 302e (Graz, Austria). The instrument was equipped with a parallel-plate measuring system, utilizing metallic disks with a defined diameter of 25 mm. A separation gap of 0.3 mm between the plates and a shear frequency of 1 Hz were maintained throughout all experimental runs, which were performed at a constant RT of 21 °C. With this analysis, viscosity values were estimated in a range of shear rate between 0.1 and 1000 s^−1^. Subsequently, to validate the suitability of the prepared formulations for vat photopolymerization techniques, the experimental viscosity curves were compared with established literature benchmarks for Digital Light Processing (DLP) and Stereolithography (SLA).^15^ Optimal printability was assessed by verifying if the recorded viscosities fell within the target processing window of 0.2 to 10 Pa s at shear rates between 5 and 20 s^−1^.

#### Fourier transform infrared spectroscopy (FT-IR)

The photopolymerization progress was monitored in real-time using Fourier Transform Infrared (FT-IR) spectroscopy. Measurements were carried out on a Thermo Scientific Nicolet iS50 spectrometer (Thermo Fisher Scientific, Norwalk, CT, USA), with data acquisition and spectral analysis managed through the OMNIC software suite (Thermo Fisher Scientific). For the samples’ preparation, the liquid monomeric mixtures were deposited onto a Si substrate, ensuring a uniform film thickness of approximately 12 μm. UV irradiation was provided by a Lightningcure LC8 broad-spectrum lamp (Hamamatsu Photonics) centered at 365 nm, operating at a controlled intensity of 400 mW cm^−2^. Spectroscopic data were collected across the mid-infrared region (4000–400 cm^−1^) with a resolution of 4 cm^−1^. To accurately track the reaction progress, spectra were recorded at predefined time intervals (0, 2, 5, 10, 20, 30, 60, 90, 120, and 180 s). The degree of monomer conversion was quantitatively evaluated by monitoring the progressive decrease in the absorption area of the reactive acrylate bands located at 1615 and 1640 cm^−1^. To account for potential variations in sample thickness and ensure reliable normalization, the peak centered at 1510 cm^−1^ associated with C=C stretching from the aromatic ring was used as an internal reference, as considered photochemically stable. All analytical measurements were performed in triplicate to guarantee statistical reproducibility.

Monomer conversion as a function of exposure time was calculated according to eqn (1).^35^1
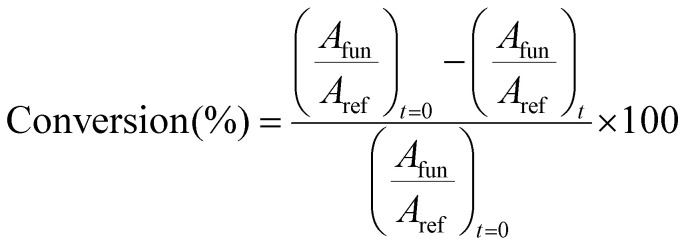


Where *A*_fun_ is the area of the two close peaks at 1615 and 1640 cm^−1^ related to the acrylate group, while *A*_ref_ is the area of the reference group at 1510 cm^−1^.

Furthermore, the rate of polymerization (*R*_p_, s^−1^) was calculated using eqn (2) ^35^ by evaluating the changes in the normalized peak areas over the defined time intervals.2
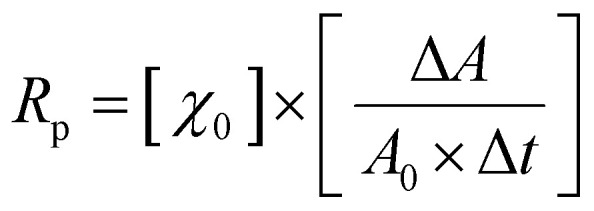


Where [*χ*_0_] is the molar fraction of acrylate monomers (in this work, [*χ*_0_] = 1), *Δ*_A_ is the difference between two subsequent areas related to the acrylate group at 1615 and 1640 cm^−1^, *A*_0_ is the initial area at a time equal to 0 s, and *Δ*_*t*_ is the time difference between two analyses.

#### Photo-differential scanning calorimetry (Photo-DSC)

To investigate the thermodynamics and reaction kinetics of the photopolymerization process, Photo-Differential Scanning Calorimetry (Photo-DSC) was performed using a Mettler Toledo DSC-1 module (Milano, Italy) equipped with a Gas Controller GC100. UV irradiation was delivered into the calorimetric chamber *via* an optical fiber connected to a Hamamatsu Lightningcure LC8 lamp, set to light intensity of 200 mW cm^−2^. Liquid resin samples, weighting between 8 and 12 mg, were placed into standard 40 μL aluminum crucibles, while an empty crucible of the same type was used as the reference. All measurements were conducted under isothermal conditions at 25 °C and a nitrogen flow rate of 40 mL min^−1^. To obtain the pure exothermic photopolymerization curve, the samples were initially allowed to equilibrate in the dark for 2 minutes, followed by a first UV exposure phase lasting 3 minutes to drive the cross-linking reaction. After an additional dark settling period of 2 minutes, a second 3 minutes irradiation was applied to the cured specimen, thus obtaining a second exothermic curve that was mathematically subtracted from the first one. It is also possible to evaluate the rate of polymerization (*R*_p_, s^−1^), by using eqn (3) ^35^ and assuming that the heat released by the samples during the analysis originates solely from the photopolymerization process.3
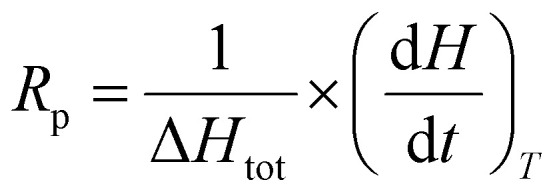


Where Δ*H*_tot_ is the total heat released during the photopolymerization (J g^−1^), calculated as the integral of the exothermic curve, and d*H*/d*t* is the heat flow measured under isothermal conditions (25 °C) (J g^−1^ s^−1^).

#### Printability tests

To optimize the vat photopolymerization process, the depth of cure (*C*_d_) and the critical exposure energy *E*_C_ were experimentally determined. The tests were carried out directly on the resin vat of a Phrozen Sonic Mighty 4K 3D printer (405 nm LED wavelength) with the build platform temporarily removed. A certain amount of each liquid resin was deposited at the center of the vat and exposed to UV light with different irradiation times (30, 40, 50, 60, 70, 80, 90 s) at RT. The resulting cured specimens (square shaped 20 × 20 mm^2^) were carefully removed from the vat and washed with ethanol in an ultrasonic bath to dissolve any residual unreacted monomer. The thickness of the polymerized films, defined as the cure depth (*C*_d_), was quantified using a micrometer. To ensure dimensional uniformity, the thickness of each sample was averaged across three distinct surface points. The total exposure energy (*E*) delivered to the irradiated geometry was calculated as the product of the light intensity (*I*, measured in 1.9 mW cm^−2^ and assumed as constant) and the specific irradiation time (*t*). By plotting the measured cure depths *vs*. the corresponding exposure energies, the characteristic Jacobs working curves were generated. According to the standard photopolymerization model expressed in eqn (4),^35^ the *E*_C_ required to obtain gelation was extrapolated from the energy intercept. Furthermore, *D*_p_ which represents the resin depth at which the incident irradiance attenuates to *e*^−1^ (approximately 37%) of its surface value, was derived from the linear slope of the curve.4
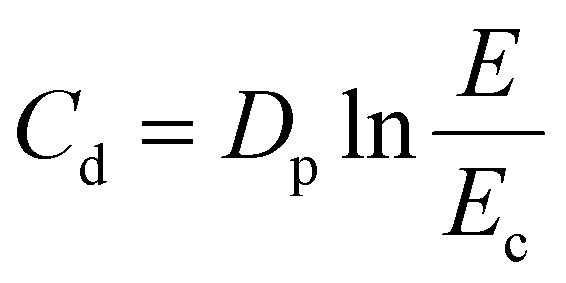


By utilizing the optimized printing parameters obtained as described above, the printability of the formulations was assessed by fabricating pinecone and honeycomb shaped 3D structures. The printing profile was configured with a layer thickness of 50 μm. Upon printing, each sample was carefully detached from the printing platform and thoroughly washed with ethanol in an ultrasonic bath for 5 minutes and after drying they were post cured for 10 minutes inside a dedicated Phrozen curing station.

#### Thermomechanical characterization

The thermomechanical behaviour and viscoelastic properties of the photopolymerized networks were characterized *via* Dynamic Mechanical Thermal Analysis (DMTA) employing a Tritec 2000 instrument (Triton Technology). For each formulation, three distinct 3D-printed samples were analyzed after undergoing a UV post-curing cycle of 5 minutes per each side. The obtained rectangular samples had average dimensions of 1 × 6 × 18 mm. The viscoelastic measurements were conducted in uniaxial tension mode, applying a constant dynamic frequency of 1 Hz. To capture the full thermal transition profile, a starting temperature of −60 °C was achieved by cooling the analysis chamber with liquid nitrogen, and an ending temperature of 150 °C was set. Then the specimens were heated at a constant rate of 5 °C min^−1^ until the storage modulus *E*′ stabilized, indicating that the material had fully transitioned into the rubbery plateau region.

Through the analysis, the evolution of the storage modulus *E*′ and the loss factor tan(*δ*) as a function of temperature was recorded. The glass transition temperature *T*_g_ of the cross-linked systems was conventionally assigned to the temperature corresponding to the peak maximum of the tan(*δ*) curve. Furthermore, the cross-link density (*υ*_C_) of the polymer networks was evaluated according to eqn (5).^35^5
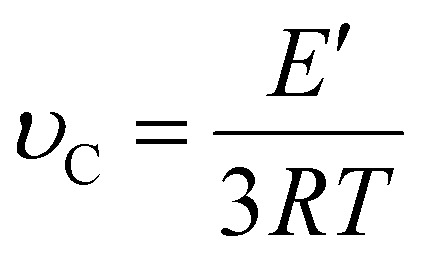


Where *E*′ is the storage modulus in the rubbery plateau registered at *T* = *T*_g_ + 50 °C (K), *R* is the gas constant (8.314 J K^−1^ mol^−1^).

#### Tensile tests

The mechanical performance of the 3D-printed formulations was assessed through uniaxial tensile tests using an MTS QTest™/10 Elite machine equipped with a 500 N load cell. All tests were carried out at a constant crosshead speed of 5 mm min^−1^ on standard dog-bone samples, which were fabricated following the ISO-527A Type 5B guidelines. Young's modulus (*E*) was calculated from the initial linear slope of the stress–strain curves, while the ultimate tensile strength and elongation were recorded at the sample's breaking point. Finally, to ensure reliable data, the reported results represent the average of three tested samples for each formulation.

#### Nuclear magnetic resonance spectroscopy (NMR)

^1^H NMR and ^13^C NMR spectra were acquired at RT in CDCl_3_ using a Bruker Ascend Evo 400 MHz and a Bruker Avance III 600 MHz spectrometers (Bruker, Karlsruhe, Germany).

## Results and discussion

### Monomer synthesis and characterization

Bisguaiacols represent a promising alternative to BPA-based printable materials. While potentially renewable and less toxic than their petrochemical counterparts,^27^ their rigid, aromatic structure imparts toughness to their deriving polymers. On the other hand, this same characteristic hinders their application in vat polymerization due to high viscosity. As a way to solve the issue while preserving the outstanding qualities of the bisguaiacol core, our approach was to impart both high toughness and flexibility by implementing a rigid, aromatic core (BGF) and flexible spacer (the hydroxyethyl ether acrylate moieties) in the monomer structure, as reported in the literature (Fig. 1).^34,36^ For the same purpose, isolation of BGF isomers was not performed, as isomeric impurity prevents stacking of phenolic cores, thus increasing the fluidity of the monomeric material. It must be noted that all materials employed in the synthesis, namely guaiacol, vanillyl alcohol, ethylene carbonate and acryloyl chloride are reported to be potentially renewable, thus making the process appealing on an environmental standpoint.^37–39^

### Rheological behaviour of photocurable formulations

**Fig. 2:**
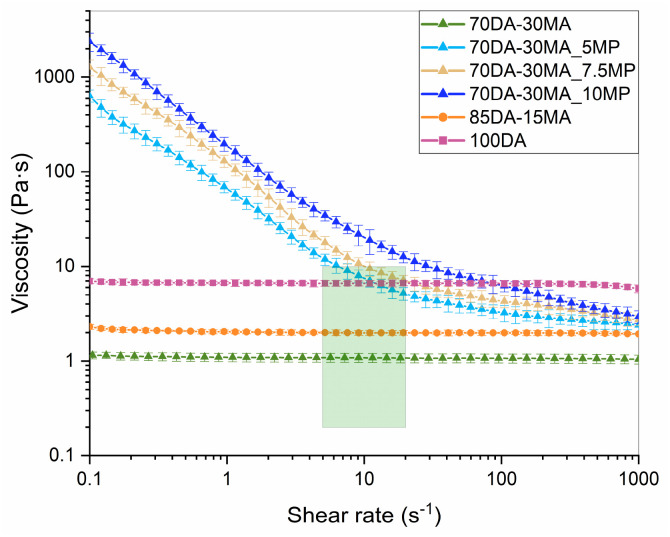
Viscosity measurements for photocurable formulations, with a green square representing the printability range for DLP/SLA 3D printing technologies.^15^

One of the most crucial parameters for vat photopolymerization 3D printing is the viscosity and rheological behavior of the photocurable formulations. To evaluate these parameters, a parallel-plate rheometer was used. In Fig. 2 the viscosity *vs*. shear rate curves are reported, obtained at RT of 21 °C for the investigated photocurable formulations. As can be seen, the viscosity of the pristine DA-MA formulation remains approximately constant across the applied shear rate range, thereby exhibiting Newtonian behavior. Furthermore, as expected, the viscosity decreases by increasing the MA reactive diluent content to the difunctional monomer DA. Conversely, following the addition of wood flour (MP), the viscosity increases with the filler content, and the rheological behaviour transitions to a non-Newtonian shear-thinning profile. This phenomenon can be attributed to the geometry of wood particles, which can be assumed as an anisotropic, highly elongated parallelepiped-like morphology, as confirmed by SEM images in Fig. S8. Under these conditions, the particles significantly hinder the movement of the monomers at low shear rates. However, as the magnitude of the applied shear increases, the particles progressively align themselves in the direction of the flow, consequently reducing the overall resistance to motion.

The green rectangle in Fig. 2 illustrates the printability window commonly accepted and reported in literature for DLP/SLA technologies,^15^*i.e.*, a viscosity of 0.2–10 Pa s at a shear rate of 5–20 s^−1^. This viscosity range assures the proper formulation spreads in the vat between each irradiation cycle with a smooth flow, enhancing the homogeneity of the print. It is evident that all three formulations without MP fall within this optimal printability range. The 70DA-30MA formulation remains suitable for printing up to a wood flour addition of 7.5 phr, whereas at 10 phr its viscosity results too high. The addition of 5 phr of MP causes the 85DA-15MA formulation to deviate significantly from the ideal viscosity range. Therefore, this formulation, alongside the even more viscous 100DA mixture, was later studied without MP addition. In contrast, all formulations falling within the printability range were fully characterized, and it was decided to also include the 70DA-30MA_10phr formulation, as its viscosity lies very close to the upper acceptable threshold.

### UV curing process

**Fig. 3:**
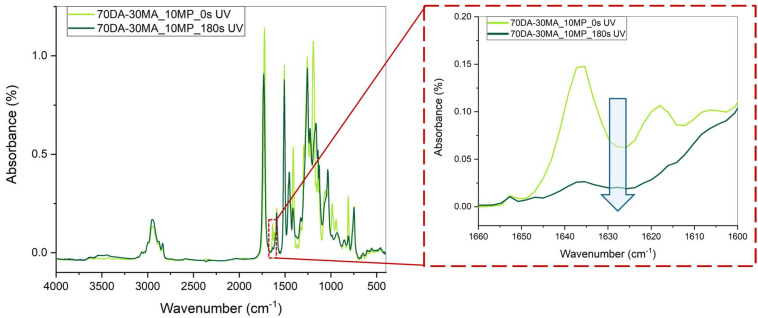
FT-IR spectra of one representative 70DA-30MA_10MP formulation before and after UV irradiation with a magnification of the decreasing peak of acrylic groups.

**Fig. 4:**
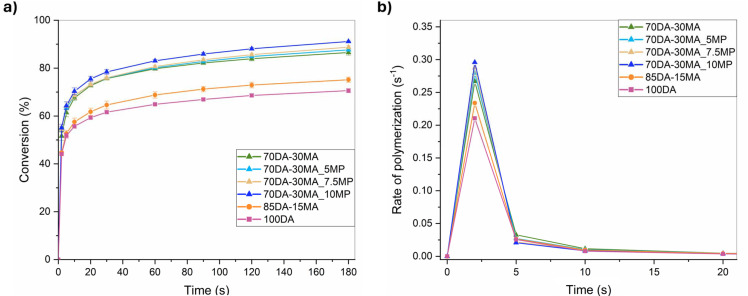
Conversion curves (a) and rate of polymerization curves (b) as a function of time for all the investigated photocurable formulations.

The UV-curing process of the different formulations was investigated by means of FT-IR analysis, by monitoring the time-dependent reduction in the intensity of the acrylic group peak centered at 1640 cm^−1^ and utilizing the ester group peak at 1800 cm^−1^ as an internal reference. As an example, in Fig. 3, the FT-IR spectra of the 70DA-30MA_10MP formulation before and after 180 seconds of irradiation are reported. The conversion curves for all the investigated formulations are collected in Fig. 4, together with the photopolymerization rate as a function of time.

The data are collected in Table 2, in which it can be seen that by increasing the reactive diluent (MA) content in the photocurable formulations, higher conversions and higher polymerization rates were reached. This behavior can be explained by a viscosity reduction within the formulations, which enhances the mobility of the polymer chains. This increased mobility facilitates molecular interactions during the photocrosslinking process.^15^ Regarding the polymerization kinetics, the reaction rate increases proportionally with the MA concentration. However, across all tested formulations, the maximum rate peak is consistently observed within the initial 10 seconds of irradiation, followed by a gradual reduction until a plateau is reached, indicating a rapid photopolymerization.

**Table 2:** Average max conversion values with standard deviation and maximum polymerization rate values obtained from FT-IR analysis, and photo-DSC results obtained from heat flow curves

Formulation	FT-IR max conversion (%)	FT-IR *R*_pmax_ (s^−1^)	*h*_peak_ (W g^−1^)	*t*_peak_ (s)	Enthalpy (J g^−1^)	Photo-DSC *R*_pmax_ (s^−1^)
100DA	71 ± 2	0.236 ± 0.002	11 ± 1	7.3 ± 0.6	206 ± 4	0.057 ± 0.008
85DA-15MA	75 ± 1	0.115 ± 0.002	13 ± 2	6.7 ± 0.6	237 ± 5	0.063 ± 0.001
70DA-30MA	87 ± 2	0.267 ± 0.006	14 ± 1	6.7 ± 0.6	253 ± 2	0.058 ± 0.001
70DA-30MA_5MP	88 ± 1	0.273 ± 0.004	14 ± 1	6.7 ± 0.6	241 ± 1	0.054 ± 0.003
70DA-30MA_7.5MP	89 ± 1	0.274 ± 0.009	15 ± 1	6.3 ± 0.6	230 ± 1	0.058 ± 0.006
70DA-30MA_10MP	91 ± 1	0.278 ± 0.004	15 ± 2	6.7 ± 0.6	223 ± 3	0.063 ± 0.004

Notably, the conversion and polymerization rate curves remain largely unaffected by the incorporation of MP up to a concentration of 10 phr. This was quite unexpected since the inherent UV-absorbing tendency of the wood flour attributed to its retained lignin fraction should typically compete with the photoinitiator and thereby hinder overall conversion.^30^ As a matter of fact, this was evidenced in the following Photo-DSC analysis, and the difference could be attributed to a difference of thickness. When 12 µm film thickness is irradiated in FT-IR analysis, the presence of the filler did not significantly hinder the UV-light penetration and therefore similar conversion and rate of photopolymerization was measured.

**Fig. 5:**
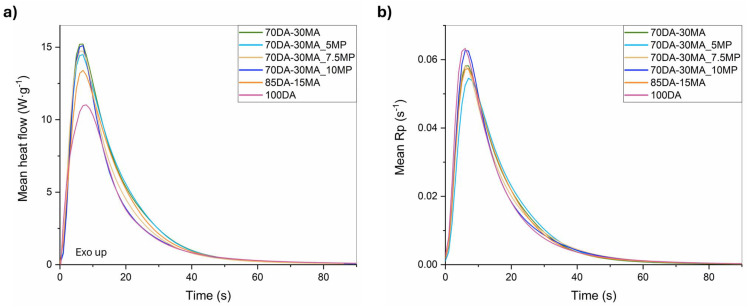
Heat flow *vs*. time curves (a) and rate of polymerization curves (b) for all the formulations.

As just mentioned, the formulations were further characterized *via* Photo-DSC. The resulting mean exothermic curves of the photopolymerization process, alongside the corresponding mean polymerization rate profiles (*R*_p_), are presented in Fig. 5. The mean values with corresponding standard deviations for several key parameters derived from the enthalpy curves are collected in Table 2. In which are summarized: the maximum heat flow peak (*h*_peak_), the time required to reach the peak maximum (*t*_peak_), the total released enthalpy calculated as the integral of the curves (Δ*H*_tot_), and the maximum photopolymerization rate (*R*_pmax_).

By comparing these results, it can be noticed that the total released enthalpy increases with the reactive diluent (MA) content, from 206 ± 4 J g^−1^ of 100DA to 253 ± 2 J g^−1^ of 70DA-30MA, thanks to the lower viscosity that leads to an easier interaction between the acrylic groups involved in the photopolymerization. On the other hand, the incorporation of the filler (MP) induces a lowering of the heat release, when compared to the 70DA-30MA pristine formulation.

This effect can be attributed to two main factors. In Photo-DSC analysis, the specimens are significantly thicker than those used in FT-IR measurements, in which the samples consist of ∼12 µm-thick films. Under these conditions, the inherent UV absorption of the wood flour can attenuate light penetration, thereby limiting monomer conversion in the deeper resin layers and consequently reducing the overall heat released. Furthermore, the enthalpy released during the cross-linking of methacrylic groups is intrinsically lower than that of acrylic groups.^40^ As a result, the formation of covalent bonds between the emerging polymer network and the filler fails to compensate for the enthalpy loss caused by the reduced conversion of the acrylic monomers.

### Determination of printing parameters by working curves

**Fig. 6:**
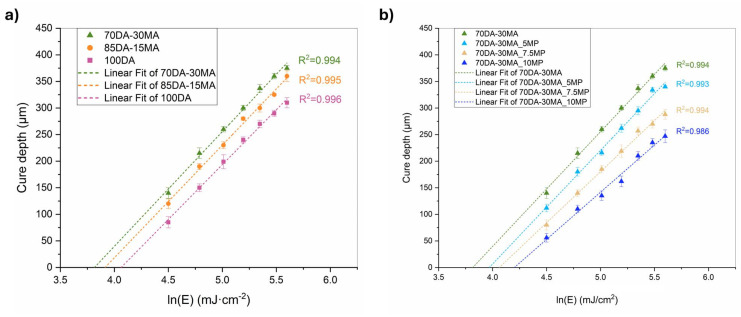
Jacob's working curves built using 3D printer of (a) DA-MA formulations without MP and (b) MP filled formulation with respect to pristine 70DA-30MA.

Jacobs working curves (Fig. 6) establish a direct relationship between the exposure time of the formulations and the resulting photocured thickness. From this analysis, several key parameters for the 3D printing process can be determined (see Table 3). In particular, the *x*-intercept of the linear regression of cure depth data represents the critical energy (*E*_C_), which is the minimum energy required for the resin to reach its gel point. The corresponding critical time (*t*_0_) is calculated using eqn (4). This equation also enables the determination of the exposure time needed to cure a 50 µm thick layer (*t*_50_), which was then selected as the standard layer thickness for the 3D printing experiments.

**Table 3:** Cure depth measurements main results

Formulation	*D*_p_ (μm)	*E*_C_ (mJ cm^−2^)	*t*_0_ (s)	*t*_50_ (s)	Irradiation time normal layers (s)	Irradiation time bottom layers (s)
100DA	206	58.1	19	25	40	60
85DA-15MA	212	50.1	17	21	35	55
70DA-30MA	216	45.4	15	19	20	40
70DA-30MA_5MP	214	52.9	18	22	30	50
70DA-30MA_7.5MP	192	58.1	19	25	—	—
70DA-30MA_10MP	177	66.8	22	29	45	60

Recalling that viscosity investigations showed that the 70DA-30MA formulation remains suitable for printing up to a wood flour addition of 7.5 phr, we have measured the Jacobs curves for these filled formulations. By analyzing the obtained results, it can be noted how the formulations containing wood flour (Fig. 6b) require longer irradiation times to achieve the same desired thickness when compared to the pristine 70DA-30MA formulation. Additionally, a reduction in the UV light penetration depth (*D*_p_) is observed at higher MP concentrations, both these phenomena are due to the inherent UV absorbing properties of the filler, as previously discussed. Furthermore, an increase in the critical energy is observed at higher wood flour concentrations. It can be attributed to the higher thermodynamic stability of the methacrylic groups characterizing the filler functionalization, which now actively integrates into the polymer network.

In contrast, comparing the unfilled formulations with different amounts of reactive diluent (MA) shows that the UV light penetration depth remains nearly unchanged across all three systems. However, decreasing the MA content slightly reduces the overall photopolymerization efficiency. This is reflected in an increase in the critical energy (*E*_C_), meaning that formulations with a higher proportion of DA require longer UV exposure to achieve the same layer thickness. Since the DA monomer and MA diluent are structurally very similar, this reduced curing efficiency is most likely due to limited molecular mobility caused by the higher viscosity of the formulations.

### 3D printing

**Fig. 7:**
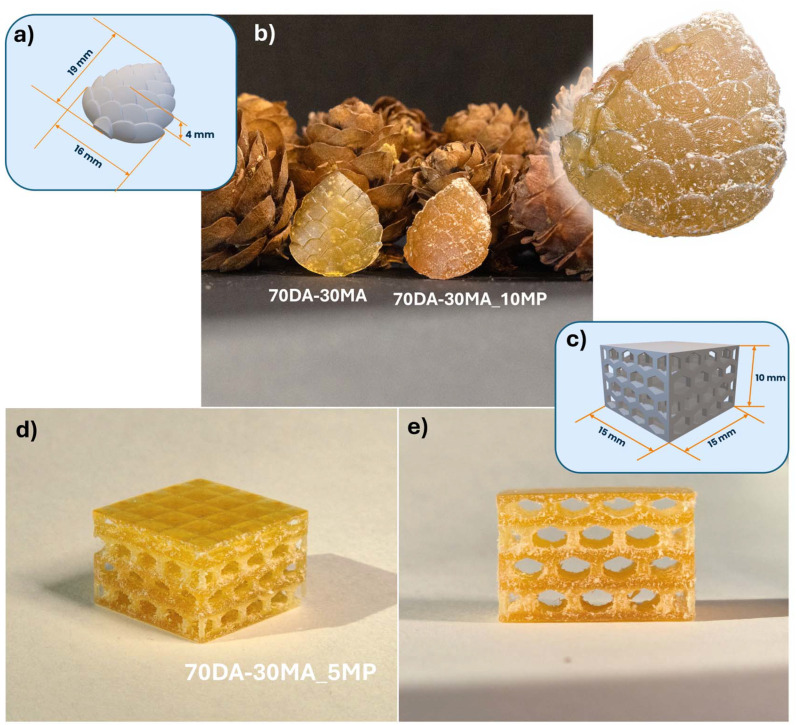
Images of the CAD files of the pinecone (a) used to 3D print with: 70DA-30MA and 70DA-30MA_10MP formulations (b), and the hollow shape (c) used to 3D print with 70DA-30MA_5MP formulation (d & e).

**Fig. 8:**
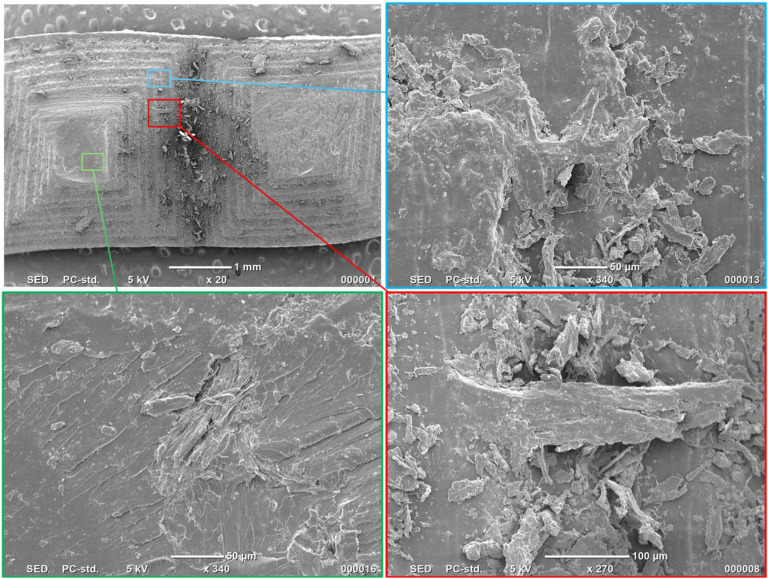
SEM images of different points on the cross-section of 70DA-30MA_5MP 3D printed sample.

During 3D printing trials, the irradiation time for each 50 μm layer was increased by around 10 s with respect to *t*_50_ values obtained from Jacobs working curves, to ensure sufficient cross-polymerization between two subsequent layers along the *z*-axis. Furthermore, the irradiation times of the first 5 layers were increased even more, to enhance their attachment to the building platform. Using the parameters listed in Table 3, a complex hollow structure was successfully 3D printed with the 70DA-30MA_5MP formulation. The 70DA-30MA_10MP formulation also produced a successful print; however, due to its higher viscosity, a simpler pinecone-shaped model was selected. The resulting 3D-printed objects are shown in Fig. 7. To fully understand the interfacial interaction between the filler and the polymer matrix, SEM images were acquired from the brittle fracture surfaces of the 3D-printed 70DA-30MA_5MP sample. As evidenced by the images reported in Fig. 8, the filler particles are deeply embedded within the matrix and remain largely coated by the polymeric phase, showing a good compatibility between the matrix and the filler, which could be assured by the presence of the methacrylic functional groups on the MP surface. Furthermore, the complete absence of observable pull-out voids serves as a strong indicator of the excellent interfacial adhesion established between the methacrylated wood flour and the surrounding polymer matrix.

**Fig. 9:**
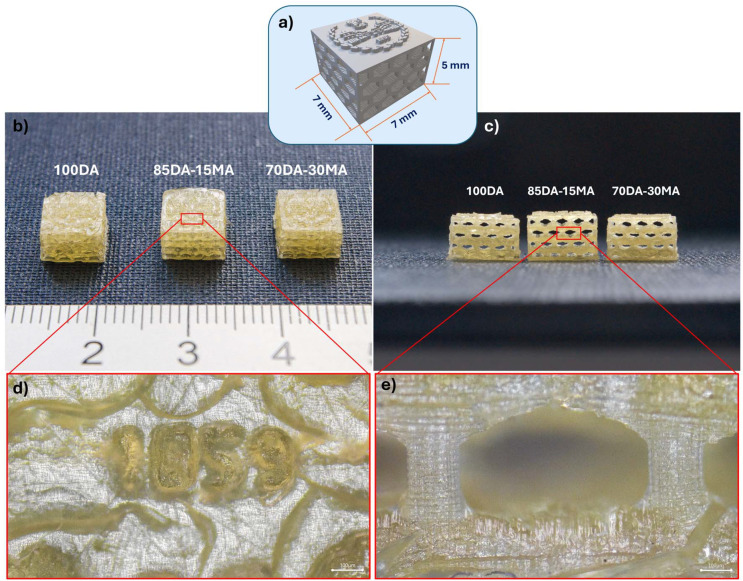
Images of the CAD file of the hollow shape (a) used to 3D print with: 100DA, 85DA-15MA and 70DA-30MA formulations (b & c); detail from the Politecnico di Torino logo on the top surface (d) and from the side of a sample (e).

To show the printability of unfilled formulations, using the parameters listed in Table 3, the complex hollow structure was successfully 3D printed, as can be seen in Fig. 9. This time the 3D model was reduced in size and decorated by adding the Politecnico di Torino logo on the top surface.

### Thermomechanical and mechanical properties

**Fig. 10:**
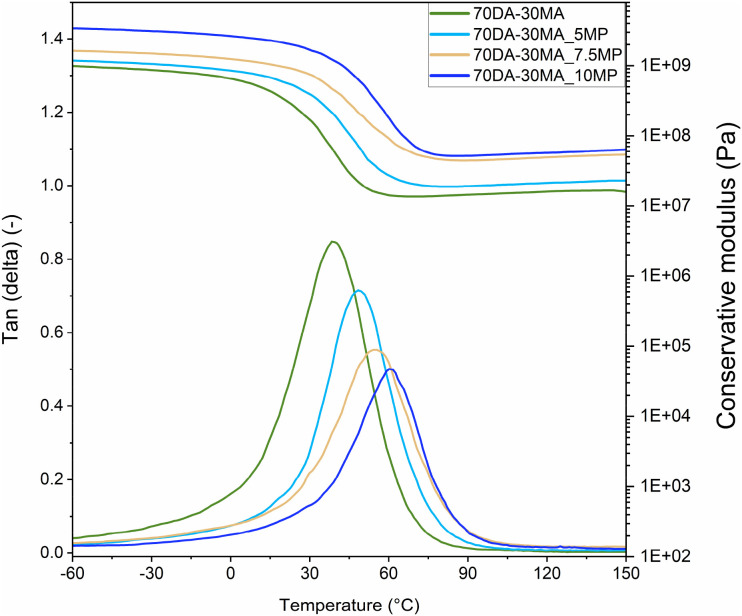
Storage modulus and tan(*δ*) *vs*. temperature curves for all the 70DA-30MA formulations, respectively in the upper and lower part of the image.

To investigate the influence of filler incorporation, the formulations containing different concentrations of MP were characterized *via* DMTA and compared against the pristine 70DA-30MA. Table 4 summarizes the average glass transition temperature (*T*_g_), determined from the peak maximum of the tan(*δ*) *vs*. temperature curves, alongside the average cross-link density calculated in the rubbery plateau region using eqn (5). The corresponding tan(*δ*) and storage modulus curves as a function of temperature are plotted in Fig. 10.

**Table 4:** Results of DMTA analysis with crosslinking densities and of tensile tests

Formulation	*T*_g_ (°C)	Storage modulus at *T*_g_ + 50 °C (MPa)	*υ*_C_ (mol L^−1^)	Young's modulus (MPa)	UTS (MPa)	Elongation at break (%)
70DA-30MA	38 ± 2	14 ± 2	1.55	22 ± 3	13 ± 3	5 ± 1
70DA-30MA_5MP	48 ± 2	19 ± 4	2.05	24 ± 6	17 ± 4	6 ± 2
70DA-30MA_7.5MP	55 ± 2	46 ± 4	4.88	31 ± 5	25 ± 1	7 ± 1
70DA-30MA_10MP	62 ± 2	56 ± 3	5.83	36 ± 2	32 ± 2	7 ± 1

By evaluating the experimental data, a notable increase in *T*_g_ following the addition of the filler can be observed, rising from 38 ± 2 °C of the pristine 70DA-30MA to 62 ± 2 °C of the 70DA-30MA_10MP composite. This shift towards a higher temperature is a direct consequence of the interaction of the polymer network with the functionalized filler, which restricts polymer chains mobility.^40^ The observed decrease in the tan(*δ*) peak height reflects the constrained conformational mobility of the polymer chains near filler particles, thus reducing internal viscous dissipation.^41^

Furthermore, by utilizing vat photopolymerization 3D printing to build the test specimens, the risk of the wood flour hindering the complete photocrosslinking of the samples is effectively minimized, as the material is cured for 50 µm thick layers at a time. Following the addition of MP, an increase in the overall crosslinking density is also observed. This enhancement can be attributed to the methacrylic functionalization of the filler, which allows it to serve as a cross-linking junction point between multiple polymer chains.

**Fig. 11:**
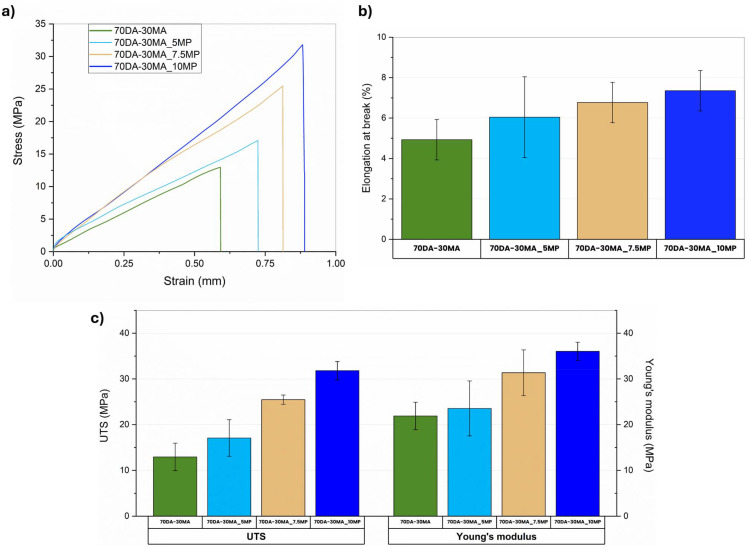
Stress–strain curves (a), mean elongation at break (b), mean UTS and mean Young's modulus (c) for all the 70DA-30MA formulations.

The mechanical properties were determined and the most representative stress–strain curves are reported in Fig. 11. The average mechanical properties extracted from these tests are graphically reported in the same figure and summarized in Table 4. As evidenced by the tabulated data, the incorporation of MP induced a distinct reinforcing effect when compared to the pristine 70DA-30MA matrix. This enhancement translates into elevated values for both the Young's modulus and the Ultimate Tensile Strength (UTS), on the other hand, the elongation at break remains largely unaffected. The observed increase in Young's modulus can be related to the higher crosslinking densities achieved by adding the methacrylated filler, as discussed for the *T*_g_ enhancement. Furthermore, the increase in UTS is a direct consequence of the excellent interfacial interaction between the filler and the polymer matrix, ensured by the covalent bonds formed through the functionalization of the wood flour, which ultimately facilitates a highly efficient stress transfer mechanism across the composite interface.^32^

## Conclusions

A fully bio-based photocurable system based on Bisguiacol-F-Diacrylate (DA) was successfully designed for vat photopolymerization 3D printing (SLA/DLP). Several formulations were prepared based on DA, with Acrylated Guaiacol (MA) as a reactive diluent. To obtain a high-performance “all-wood” polymer composite Methacrylated Pinewood flour (MP) was dispersed as a filler in the photocurable formulation up to a content of 10 phr. Rheological analysis demonstrated that optimizing the DA/MA ratio (70DA-30MA) allowed for an effective modulation of the pristine viscosity, ensuring excellent printability at RT and enabling the incorporation of the filler up to a maximum of 10 phr. Reaction kinetics studies (*via* FT-IR and Photo-DSC) confirmed that the inherent UV-absorbing capability of the wood flour did not compromise significantly the process efficiency. The formulations maintained rapid polymerization rates and high monomer conversion degrees (up to 91% after 180 s of UV irradiation), allowing for the fabrication of complex, high-resolution 3D geometries. From a thermomechanical and structural perspective, the results confirmed the crucial role of the filler's functionalization. Morphological analyses (SEM) of the fracture surfaces of the printed samples revealed excellent interfacial adhesion, without any signs of pull-out voids, demonstrating the successful covalent bonding between the methacrylic groups of the wood flour and the polymer network. This strong synergy translated into a neat increase in the glass transition temperature (*T*_g_), which enhanced from 38 °C for the pristine 70DA-30MA formulation to 62 °C for the composite with 10 phr of MP. At the same time, a notable increase in Young's modulus and ultimate tensile strength (UTS) was measured, while the elongation at break remained largely unaffected.

In conclusion, this work demonstrates the concrete feasibility of replacing conventional fossil-based aromatic monomers with biomass-derived aromatic alternatives for vat 3D printing. The combination of functional monomers and reactive fillers easily obtained from wood represents a highly effective strategy to bridge the gap between environmental sustainability and engineering performance, opening the way for the next generation of renewable photopolymers and advanced composites.

## Author contributions

E. G. conducted experimental analysis. N. P. performed monomer syntheses, purification and characterization. M. B. performed rheological characterization. N. B. and F. R. supervised the work performed at the University of Modena and Reggio Emilia and corrected the paper. M. H. prepared the methacrylated wood flour and corrected the paper. M. S. supervised the work, financed the work and corrected the paper.

## Conflicts of interest

There are no conflicts to declare.

## Supplementary Material

PY-017-D6PY00486E-s001

## Data Availability

The datasets generated during and/or analysed during the current study are available from the authors on reasonable request. Supplementary information (SI) is available. See DOI: https://doi.org/10.1039/d6py00486e.
