# Supplementary material for: From trees to 3D printing: “all-wood” photopolymer composites based on bisguiacol-F-diacrylate and methacrylated pinewood flour for digital light processing (DLP)
Source: Polym Chem. 2026 Jun 11;17(26):2814–27. doi: 10.1039/d6py00486e (PMC13254514; doi:10.1039/d6py00486e)
Supplement: PY-017-D6PY00486E-s001 [file PY-017-D6PY00486E-s001.pdf]

Supplementary Information (SI) for Polymer Chemistry.
This journal is © The Royal Society of Chemistry 2026

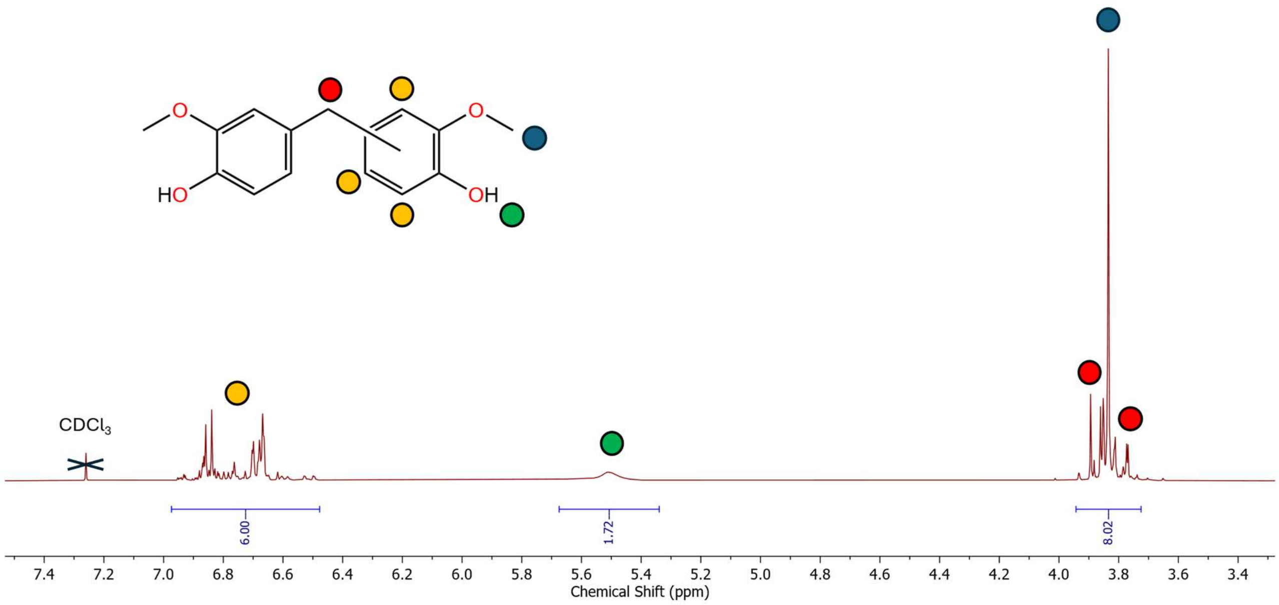

Figure S1 ^{1}H NMR of **BGF** (bisguaiacol F) with peak assignments. 600 MHz, $CDCl_3$, δ (ppm): 6.97– 6.47 (m, 6H, Ar-H), 5.67-5.34 (bs, 2H, Ar-OH), 3.94-3.0.72 (m, 8H, Ar-O-CH_3 and Ar-CH_2-Ar).

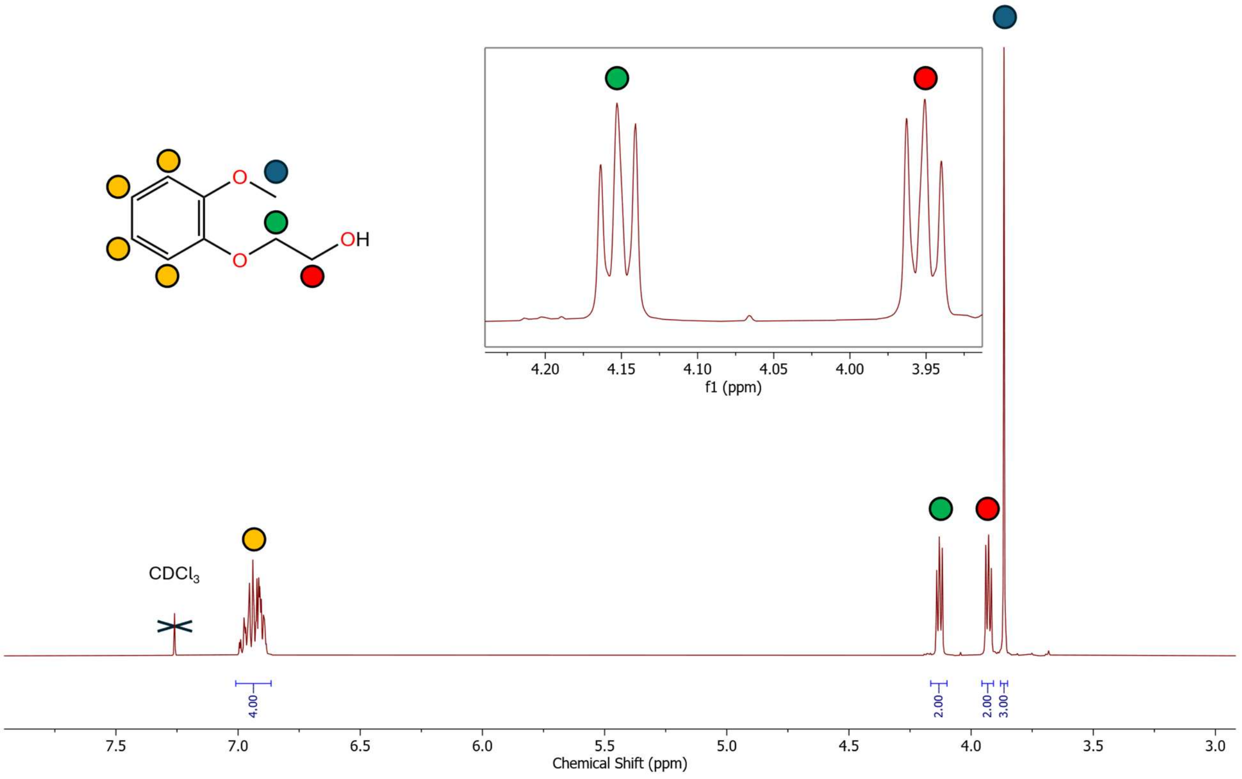

Figure S2 ^{1}H NMR of **GuaEG** with peak assignments. 400 MHz, $CDCl_3$, δ (ppm): 7.00– 6.87 (m, 4H, Ar-H), 4.12 (t, J = 4.4 Hz, 3H, Ar-O-CH_2), 3.92 (t, J = 4.8 Hz, 3H, C-CH_2-O), 3.86 (s, 3H, Ar-O-CH_3).

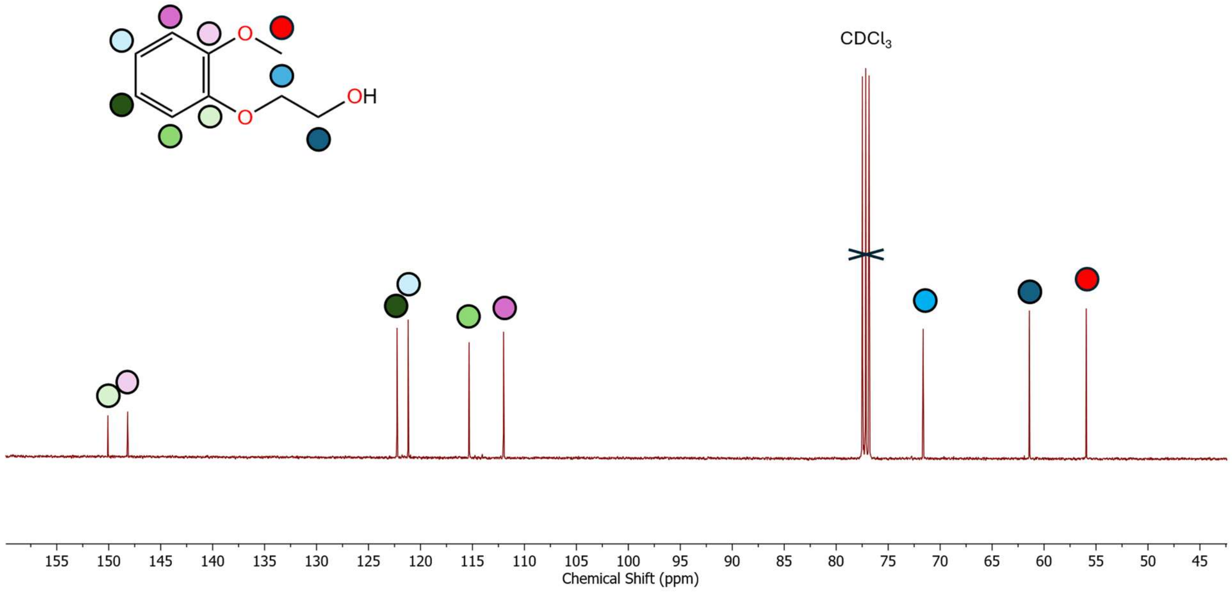

Figure S3 ^{13}C NMR of GuaEG with peak assignments. 100.61 MHz, $CDCl_3$, δ (ppm): 150.1, 148.2, 122.3, 121.2, 115.3, 112.0, 71.6, 61.4, 55.9.

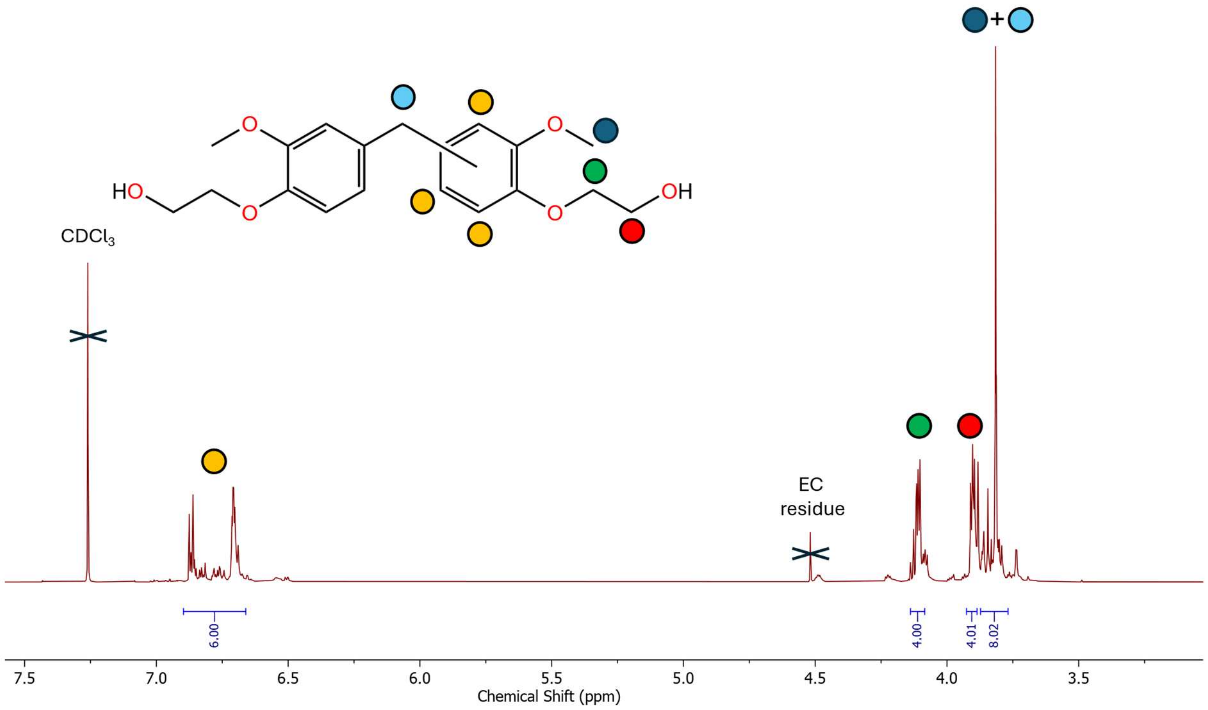

Figure S4 ^{1}H NMR of **BGFEG** with peak assignments. 400 MHz, $CDCl_3$, δ (ppm): 6.89– 6.62 (m, 6H, Ar-H), 4.12-4.09 (m, 4H, Ar-O-CH_2), 3.91-3.88 (m, 4H, C-CH_2-O), 3.88-3.72 (m, 8H, Ar-O-CH_3 and Ar-CH_2-Ar).

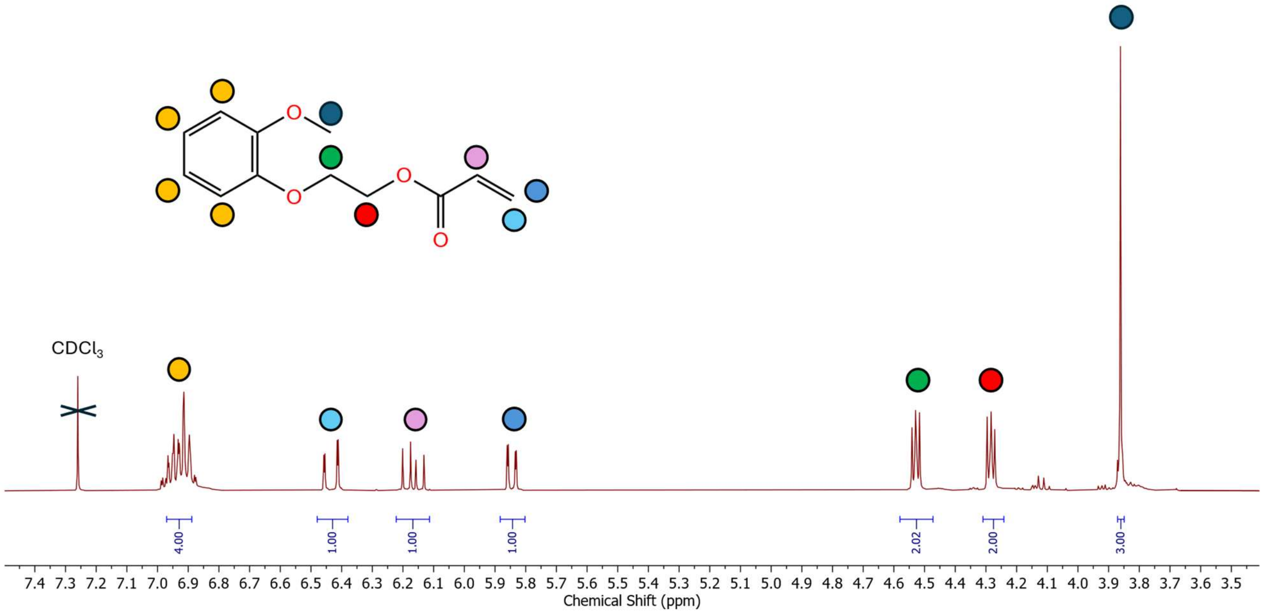

Figure S5 ^{1}H NMR of **MA** with peak assignments. 400 MHz, $CDCl_3$, δ (ppm): 7.00– 6.87 (m, 4H, Ar-H), 6.43 (dd, J1 = 1.2 Hz, J2 = 16.8 Hz, 1H, C=CH2), 6.17 (dd, J1 = 10.4 Hz, J2 = 17.4 Hz, 1H, C(=O)-CH=C), 5.84 (dd, J1 = 1.2 Hz, J2 = 10.4 Hz, 1H, C=CH_2), 4.53 (t, J = 4.8 Hz, 2H, Ar-O-CH_2), 4.28 (t, J = 5.2 Hz, 3H, C-CH_2-O-C(=O)), 3.86 (s, 3H, Ar-O-CH_3).

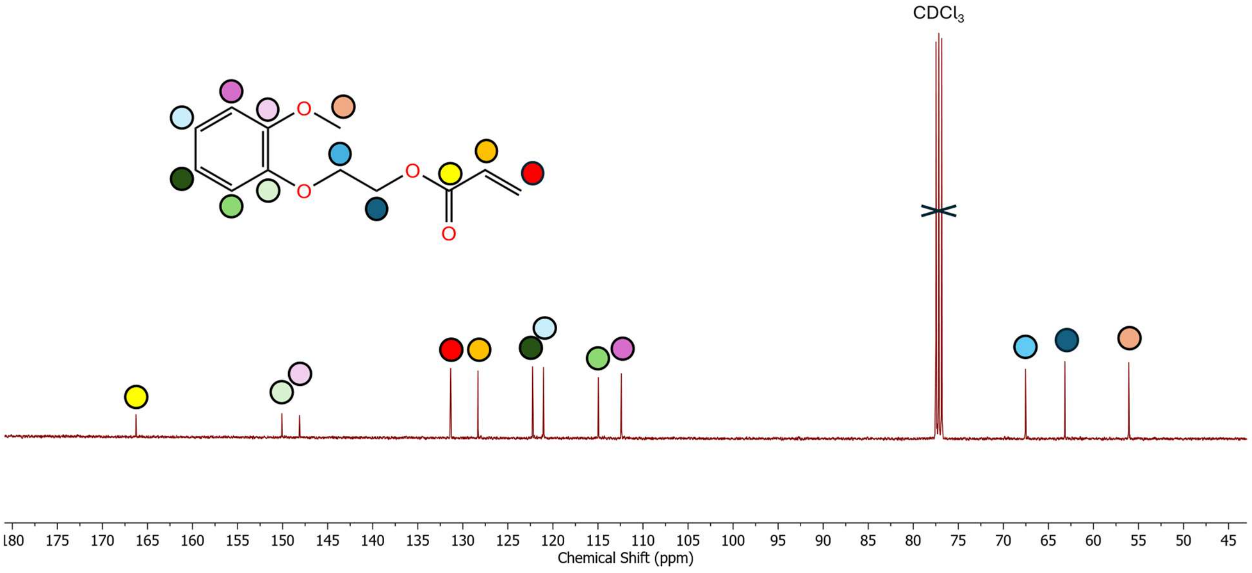

Figure S6 ^{13}C NMR of **MA** with peak assignments. 100.61 MHz, $CDCl_3$, δ (ppm): 166.3, 150.1, 148.1, 131.3, 128.3, 122.2, 121.1, 115.0, 112.4, 67.5, 63.2, 56.1.

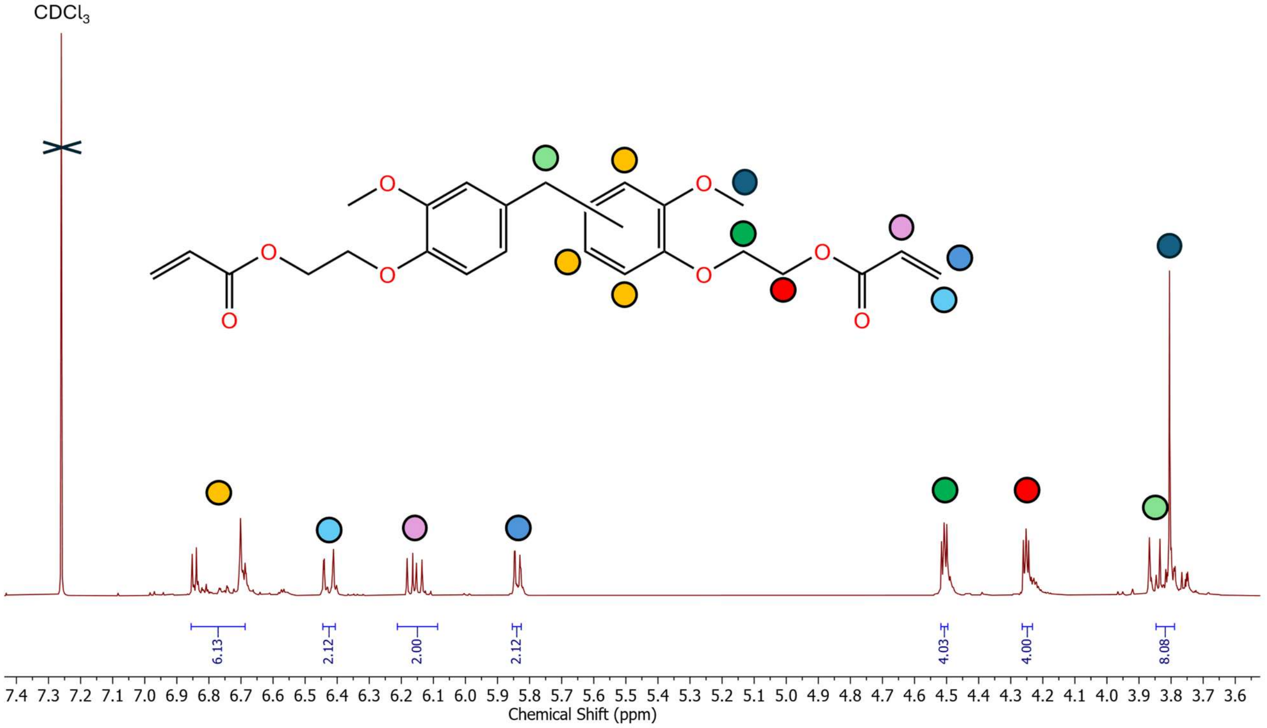

Figure S7 ^{1}H NMR of **DA** with peak assignments. 600 MHz, $CDCl_3$, δ (ppm): 6.88– 6.63 (m, 6H, Ar-H), 6.42 (dd, J1 = 1.2 Hz, J2 = 17.4 Hz, 2H, C=CH_2), 6.16 (dd, J1 = 10.8 Hz, J2 = 17.4 Hz, 2H, C(=O)-CH=C), 5.84 (dd, J1 = 1.8 Hz, J2 = 15.6 Hz, 2H, C=CH_2), 4.50 (t, J = 5.4 Hz, 2H, Ar-O-CH_2), 4.25 (t, J = 4.8 Hz, 3H, C-CH_2-O-C(=O)), 3.89-3.77 (m, 8H, Ar-O-CH_3 and Ar-CH_2-Ar).

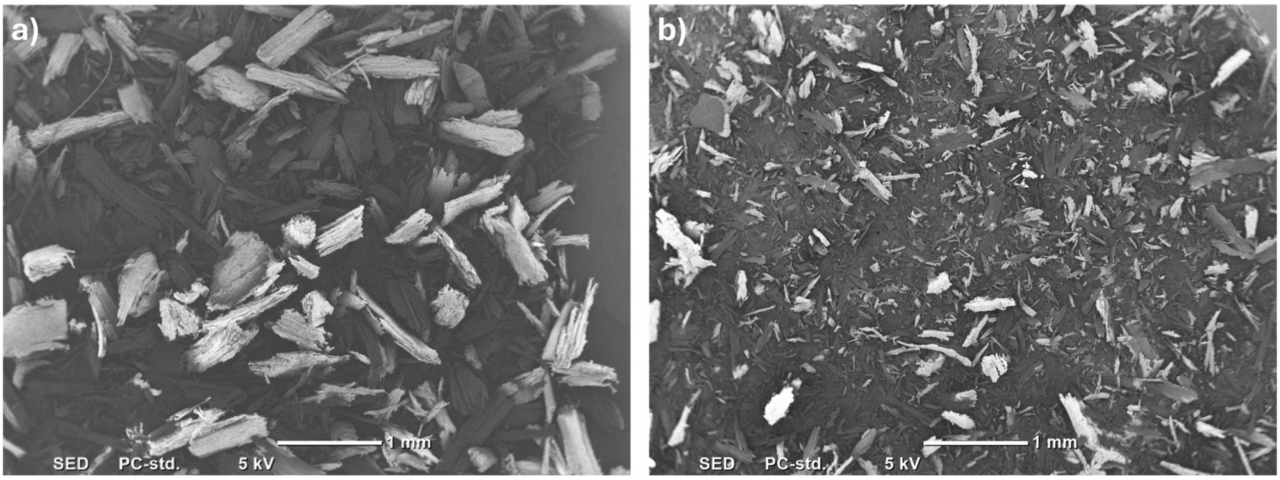

Figure S8 SEM images of MP filler before (**a**) and after (**b**) grinding.
